# What do faculty members know about universal design and digital accessibility? A qualitative study in computer science and engineering disciplines

**DOI:** 10.1007/s10209-022-00875-x

**Published:** 2022-03-29

**Authors:** Norun Christine Sanderson, Siri Kessel, Weiqin Chen

**Affiliations:** OsloMet – Oslo Metropolitan University, P.O. Box 4 St. Olavs plass, N-0130 Oslo, Norway

**Keywords:** Digital accessibility, Universal design, Digital learning materials, Higher education, Thematic analysis

## Abstract

*Purpose* Students in higher education are a diverse group comprising people with different backgrounds and abilities. Regulations require that digital learning materials and platforms employed in higher education accommodate this diversity. Furthermore, they require faculty members to have an understanding of universal design and digital accessibility, as well as practical knowledge of how to make learning materials and courses accessible for more students. The goal of this research is to gain insight into the status of such knowledge among faculty members. *Methods* The research presented in this paper involved a qualitative study. Semi-structured interviews were conducted with 35 faculty members employed in higher education institutions (HEIs) in Norway and Poland. The participants worked in the computer science and engineering disciplines. The data was analysed using thematic analysis, and two main themes and six sub-themes were identified. *Results* We found that most participants lack sufficient understanding of digital barriers and assistive technologies. Very few were aware of legislation and guidelines related to universal design. Most importantly, the majority lack practical knowledge on how to make digital learning materials and courses accessible. Furthermore, the solutions they propose for addressing the barriers are intuitive and only encompass barriers that are easy to recognise and identify. *Conclusion* The findings indicate that there is a gap between legislation and implementation in practice when it comes to making digital learning materials accessible in higher education. The lack of knowledge among faculty members shows that training is necessary to increase understanding and practical knowledge, and HEIs should prioritise this in strategies and action plans going forward.

## Introduction

The increased prevalence in many countries and education institutions of the right to equal access to education for all members of society, including people with disabilities, has led to a more diverse student group in higher education. According to the 2018 European Student Survey [1], an average of 18% of students in higher education report having a disability or chronic disease.

This shift towards a more diverse student population and the increased digitalisation of the education sector make it necessary to consider how a manifold of people can access digitally-available information, lectures and learning materials in their chosen education, as well as the information and communication technology (ICT) systems used by students in the educational institutions. As established in earlier research, accessibility barriers in learning platforms and learning materials prevent students from fully participating in higher education [2–4], thus impeding their prospects for academic achievement.

Legislation both internationally and at the national level reflects this need to consider access to education for all students, or inclusive education. The European Disability Strategy 2010–2020 [5] sets out education and training as one of its eight priority areas, forming part of its overall objective of promoting equal access to quality education and lifelong learning. Teachers play an important role in ensuring equal access to education, among other things by making digital learning materials accessible for all students. The increased focus on access to education for all and the manifestation of such in legislation, strategies and regulations is promising. However, unless teachers and faculty members are familiar with universal design of digital learning materials, and how to implement it in practice, barriers to academic success in higher education will still be present for many students. This paper therefore focuses on what faculty members know about universal design and digital accessibility.

The research presented in this paper involves a qualitative study aiming to gain insight into the attitudes, knowledge and experience of faculty members in the fields of computer science and engineering when it comes to universal design. Interviews with 35 faculty members employed in higher education institutions in Norway and Poland were analysed. Results previously published as part of this study [6] concerned an analysis of the attitudes of faculty members when it comes to making their learning materials accessible. In this paper, we focus on what faculty members know about universal design and what they need to do to ensure that their courses and digital learning materials are accessible.

The paper is organised as follows: Sect. 2 covers relevant background information related to universal design and legislation. In Sect. 3, relevant research is presented, while methods used in the study are covered in Sect. 4. Section 5 presents the results from the study, followed by a discussion of the results in Sect. 6, and conclusion and future work in Sect. 7.

## Background

### Universal design

Originating from architecture and building design in the mid-1980s [7], the concept of universal design is today considered to be a fundamental principle of good design. It signifies designing or composing (human-made) environments, buildings, products and services in such a way that they can be accessed and used to the greatest extent possible by all people [8].

The term Universal Design is commonly used in a wealth of disciplines involving design and the creation of something intended to be used by the general public, such as technology/ICT, product design and teaching disciplines, to name a few. Universal design is often used interchangeably with terms such as design for all and inclusive design. The definition adopted in Norway is stated in the Norwegian Equality and Anti-discrimination Act of 2017, Sect. 17:

“‘Universal design’ means designing or accommodating the main solution with respect to the physical conditions, including information and communications technology (ICT), such that the general functions of the undertaking can be used by as many people as possible, regardless of disability.” [9]

In this paper, we use the term Universal Design in accordance with this definition. A more proactive approach to Universal Design or Design for All is defined in Stephanidis [10] emphasising that principles, methods and tools must be proactively applied throughout the design and development life cycle.

**Fig. 1:**
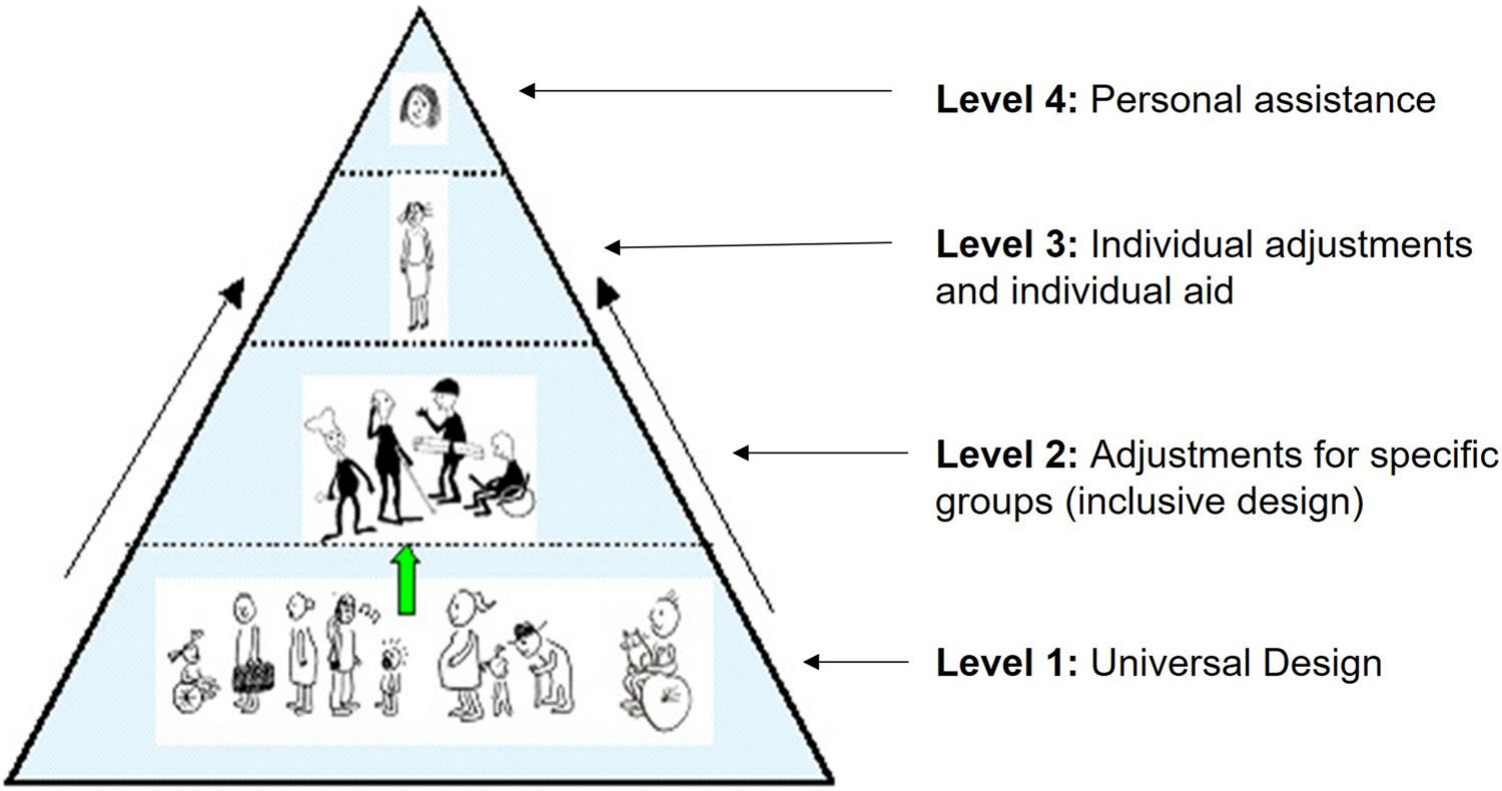
The accessibility pyramid, adapted from [11]

ICT users have varying levels of abilities, ranging from those who can use any ICT equipment without any adaptation to those who cannot use any ICT equipment without personal assistance, as reflected in the accessibility or usability pyramid [11–14]. Originally created by the Norwegian Delta Centre [11], the accessibility pyramid illustrates how systems must be designed to take into consideration different levels of accessibility. It ranges from “Universal Design” (level one), which will allow as many people as possible to use a solution without customisation or adaption, via “Adjustments for specific groups”, which also takes inclusive design or adaptations for groups into consideration, for example sign language or Braille, to “Individual adjustments and individual aid”, indicating that some may need personalised adaptation to be able to use a solution, and “Personal assistance” (level four), which includes situations where the user can only use the system with the assistance of another person. Fig. 1 shows an adapted version of the accessibility pyramid, where the levels of accessibility have been numbered for clarity, starting at the bottom with level one and moving upwards to level four at the top.

With the aim of developing a single shared standard to ensure accessible web content for people with disabilities, the World Wide Web Consortium (W3C) launched the Web Content Accessibility Guidelines (WCAG), which define a set of testable criteria that must be met to ensure equal access to web content. These guidelines are accompanied by a suite of documents providing technical advice on how to interpret the criteria, as well as how to meet them in practice. WCAG is organised into four main principles: *Perceivable*, *Operable*, *Understandable* and *Robust*. The principles each consist of a set of guidelines with testable success criteria. Each criterion corresponds to a level of conformance (A—lowest, AA, AAA—highest) indicating the impact of the accessibility issue in relation to diverse groups and situations [15]. The current version, WCAG 2.1, was approved in 2018 [16]. However, the previous version approved in 2008, WCAG 2.0, became an ISO International Standard in 2012 [17]. Since the interviews were conducted in 2016, we used WCAG 2.0 in our analysis.

### Relevant legislation

Internationally, the UN Convention on the Rights of Persons with Disabilities (UN CRPD) [18], addresses the accessibility of education in the Preamble of the Convention, while Article 24 on Education, point five, specifically addresses the responsibility to ensure equal access to “*general tertiary education, vocational training, adult education, and lifelong learning”.* Point four of Article 24 on measures towards the realisation of the right to education states that the training of professional staff at all levels of education must include “*disability awareness and the use of appropriate augmentative and alternative modes, means and formats of communication, educational techniques and materials to support persons with disabilities*”. Equal access to information and communication technologies and related systems and services is considered in Article 9 of the Convention, specifically stated in points 1 b, 2 g, and 2 h, which together cover electronic services, emergency services, the internet, as well as the design, development, production and distribution of accessible systems. Poland ratified the UN CRPD in September 2012 and Norway in May 2013 [19], and are thus obliged to implement the CRPD in their national legislation.

There are no legally binding documents concerning education at EU level since the responsibility for education lies with each member state. The EU’s function is thus to provide support and coordination [20, 21]. The Web Accessibility Directive of 2016 [22] does not specifically cover education, but encourages member states to extend the provisions to also cover websites or mobile applications used in education, and states that requirements of reasonable accommodation still apply (points 34 and 38 in the directive). Poland has been an EU member state since 2004 [23], and is thus under obligation to implement the EU Directives in its national legislation. Norway is a member of the European Economic Area (EEA) [24] and the European Free Trade Association (EFTA) [25], and must therefore harmonise national legislation relevant to access to the EU’s Internal Market [26].

At the level of national legislation, both countries had legislation that covered access to education for persons with disabilities in 2016 when our study took place. We will present relevant current legislation and governmental programmes also at this level to emphasise the ongoing developments taking place in this area.

In Norway, the first Anti-Discrimination and Accessibility Act prohibiting discrimination on the basis of disability [27] came into force in 2008 (amended in 2013), covering all aspects of society, including information and communication technology. Regulations for universal design of information and communication technology (ICT) solutions [28] took effect in July 2013, but did not at that time specifically cover ICT solutions used in education. Section 4-3(2) of the Norwegian Act relating to universities and university colleges [29] from 2005 states that it is the board of the institution that has the overall responsibility for ensuring a suitable learning environment as far possible and reasonable, and specifies in Section 4-3(2) (i) that this entails ensuring “*that the learning environment is designed according to the principles of universal design”.*

In Poland, the Law of Higher Education of 2005, with amendments in 2011, states that institutions of higher education are obliged to establish conditions ensuring the full participation of persons with disabilities in processes of learning and research, and that these are among the principal objectives of higher education institutions. It further states that study regulations should specify methods for appropriate implementation of the teaching process taking into account the needs of disabled students as well as the type of disability, as should entrance requirements and procedures [30].

## Related research

### Knowledge among faculty members

The findings of several relevant studies on the accessibility of higher education for students with disabilities indicate that instructors and faculty members lack knowledge about accessibility and how to accommodate students with disabilities in their courses. Knowledge of accessibility among faculty in technology departments may be very low indeed, particularly among those not teaching accessibility as part of their courses. Findings from a US survey by Shinohara et al. [31] on computing faculty teaching accessibility in higher education show that the majority (66.1%) of faculty teaching accessibility self-reported their knowledge of accessibility as “Some knowledge” while 10.7% considered they were “Not knowledgeable”. Only 4.5% rated their knowledge as “Expert”, and 18.7% as “Knowledgeable”.

Knowledge about disabilities and differences was considered a barrier across all participant groups in a study by Marquis and colleagues [32], both in terms of the participants’ own lack of knowledge and that of others at the university. Knowledge was also found to be one of the five main types of barriers mentioned by most participants in this study [32–34], the other four types being attitudes, pedagogical choices, disciplinary features, and institutional practices and characteristics. Many participants in the study also suggested that the lack of knowledge could be linked to attitudinal barriers. These findings are based on a three-phase qualitative study looking into the accessibility and inclusiveness of education at a Canadian university, on the basis of interviews with five groups of participants across the university representing the administration, staff, instructors and students with and without disabilities.

The lack of knowledge among instructors on how to accommodate students with disabilities in laboratory classes can be perceived as a barrier by students, even when there is a great willingness among the instructors to accommodate them. This is exemplified by findings from a nationwide survey among US students with disabilities in science and engineering education by Jeannis et al. [35], where 30.8% of the participating students perceived lack of knowledge to be a barrier, while 66.4% noted a high willingness among the instructors to accommodate disabled students.

Knowledge about universal design requirements in higher education appears to be very limited or lacking entirely, as found in a study by Proba Research [36] investigating the use of digital learning materials and knowledge about universal design, where 27 people at different levels of Norwegian higher education were interviewed. Another finding from Proba Research shows that the administrative staff working with accommodation at the institutions had more knowledge of the requirements than other staff. Overall, educational institutions in Norway appear to have little knowledge about disabilities and insufficient time to develop solutions. This is also confirmed by the findings of Langørgen et al. [37], which, based on interviews with 14 students, looks into disabled students’ experiences of higher education. Clear directions, guidelines and training to increase awareness and know-how of how to achieve universal design in practice are among the measures suggested by Proba Research to remedy this situation [36].

Other findings show that faculty members may not satisfactorily address the areas that students with disabilities consider to be important. This is indicated in a study by Cook et al. [38], investigating faculty members’ understanding and priorities regarding students with disabilities at a university in the US (8-campus system). The areas rated by participants as important but not satisfactorily addressed included issues related to law, universal design for instruction and disability characteristics. Their results also indicate that there may be little practical knowledge in the area of assistive technologies and the provision of accessible learning materials, as the two statements in their survey achieving the lowest agreement rating were related to faculty members’ familiarity with assistive technology (32% agreement index) and faculty members’ providing learning materials in different formats and media (46% agreement index).

### Policies and responsibility for training and implementation

Several studies point towards the training of instructors and staff as a promising measure to increase knowledge of accessible education [e.g. 20–22, 24, 25, 27–29], as well as having policies on this issue and a clear distribution of responsibility for ensuring their implementation in the HEI [39, 40].

Key findings, for example in Linder et al. [40] in their study of US HEIs, indicate that HEIs need to be clear on where in the institution responsibility for accessibility lies, and to make institutional investments when creating accessible online environments. According to a study by Holloway [39] investigating students with disabilities’ experiences at a UK university, there is also a need for policies that ensure an accessible learning environment, and the implementation of these policies must be coordinated centrally in the institutions. Holloway further emphasises the necessity of practical guidelines for the various departments, as well as staff training and awareness, and the need for continuous monitoring and evaluation, the latter involving the students with disabilities.

It is furthermore necessary that faculty members have specific knowledge in a number of areas, including disability characteristics, disability law and instructional techniques, as recommended by Cook et al. [38]. They also recommend that training and information for faculty members on this topic specifically address the areas in which they lack understanding rather than focusing on general concepts and attitude change.

### What influences faculty attitudes

Although it is uncertain whether knowledge directly influences attitudes in general [41, 42], cognition (knowledge) is considered one of the components of attitudes, the other components being affect (feelings) and action [43]. Furthermore, studies have found that attitudes can change with knowledge and experience [41, 43, 44], and that knowledge may be correlated with beliefs and confidence [45]. It may also influence decisions made by academic staff and professionals regarding work placements for students with disabilities in professional courses [46, 47]. Leyser and Greenberger [44] found that participants who reported either personal contact (experience) with people with disabilities and/or having had training (knowledge) in the area of disabilities, showed significantly more willingness to provide accommodations and more positive attitudes. McManus et al. [41] found that increased contact (experience) positively influenced attitudes, while their findings did not indicate that knowledge directly influenced attitudes.

## Methods

A qualitative approach was considered suitable for this study, since the main objective was to explore participants’ knowledge, attitudes and experiences at a deeper level. This requires participants to communicate their thoughts, understanding and individual know-how more freely than a typical quantitative approach would have permitted. By employing semi-structured interviews with more general and open-ended questions, this study allowed faculty members to express their opinions and experiences in their own words, thus giving greater opportunity for reflection and depth.

In all, 35 faculty members, 17 in Poland and 18 in Norway, participated in this study. They were recruited through emails to contact persons at the faculties of computer science and engineering at institutions of higher education in Poland and Norway. All persons who responded to our invitation were interviewed, and each participant received a gift card after the session. The participants from Poland were all recruited from the same university, albeit from different departments, while participants from Norway were recruited from seven different universities and university colleges in the south of Norway. The university in Poland was chosen based on many years of close cooperation with the researchers, while the institutions in Norway were chosen based on geographical distance from Oslo, allowing for a one-day trip to conduct the interviewers. The contact persons in relevant faculties in Norway were identified using the institution’s website, while recruitment in Poland was performed through our contact person at the university. The contact persons were asked to forward our invitation for participation to faculty members in relevant faculties/departments of their institution. One of the researchers was responsible for making the initial contact with the contact persons, while two hired research assistants with no prior affiliations with the contact persons were responsible for following up responses from potential participants and for conducting the interview sessions. An overview of participants’ demographics is given in Table 1.

**Table 1:** Overview of participants

	Total	Female	Male	Age range	Year range teaching
Poland	17	1	16	27–55	2–16
Norway	18	5	13	24–65	0.5–22

Most of the participating faculty members were affiliated to the departments of computer science, electronic engineering and complementary subjects. Our choice to focus recruitment for this study on technology, computer science and engineering faculties was influenced by findings from a pilot study by Black et al. [48] on faculty members’ attitudes towards students with disabilities and their readiness to accommodate these students. Their findings indicated somewhat more negative attitudes and lower comfort levels around students with disabilities among these faculties when compared with other faculties.

All of the interview sessions took place during July and August 2016 in Poland and Norway. Ahead of the data collection process, the researchers filled in an online form for the Norwegian Centre for Research Data (NSD) [49] with information about the planned data collection. The NSD evaluated that there was no need to report the project to them, based on the type of data collection (no personal data and fully anonymised). The two hired research assistants conducted the semi-structured interviews, which took place at the participants’ faculties and lasted up to 36 min, depending on how much the participant had to share on the subjects addressed in the interview. The interview guide is included in the appendix. Both research assistants had experience of interviewing participants as part of their master’s degree projects and rudimentary knowledge of interview techniques as part of a master’s degree level university course on research methods. Prior to starting the interviews, the participants filled in a consent form. The interview sessions were recorded using a digital voice recorder (Olympus WS812) and transcribed verbatim by one of the research assistants. The voice recordings were deleted after being transcribed.

Interview topics covered participants’ knowledge and thoughts on guidelines, regulations and laws related to accessibility and universal design, and their personal experiences, intentions and perceived challenges with accommodating diverse students and implementing inclusion in higher education.

Three researchers conducted a qualitative data analysis using NVivo 11 of the data imported from the transcribed interviews. After systematic coding of the textual data from the transcribed interviews, the researchers carried out a thematic analysis in three iterations focusing on attitudes and knowledge. The analysis related to attitudes has been published in Chen et al. [6], while the findings related to knowledge are presented below in the next section.

## Results

**Fig. 2:**
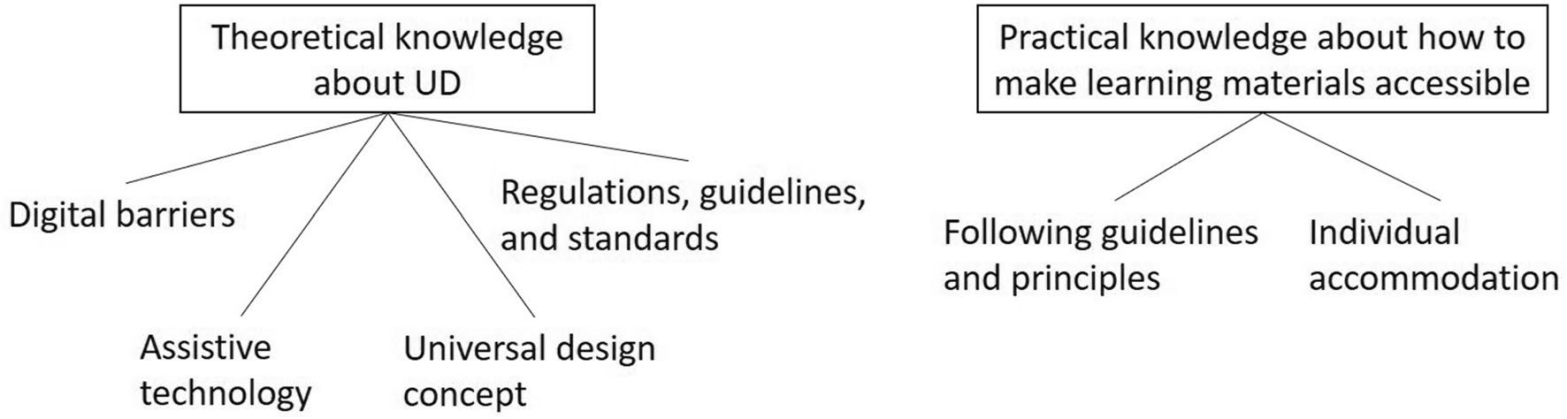
Final themes

The analysis presented in this paper focuses on what faculty members know in relation to universal design and digital accessibility, with particular emphasis on their knowledge when it comes to learning materials. Two main themes of knowledge emerged from the thematic analysis: *Theoretical knowledge about universal design*, and *Practical knowledge about how to make learning materials accessible*. The first main theme has four subthemes (knowledge topics): *Digital barriers, Assistive technology*, *Universal design concept* and *Regulations, guidelines and standards*. The second main theme has two subthemes: *Following guidelines and principles*, and *Individual accommodation.* An overview of the main themes and subthemes is given in Fig. 2.

In the following sub-sections, the two main themes with the subcategories theoretical and practical knowledge are described and presented, with examples and citations from the interviews.

### Theoretical knowledge about universal design

This section presents the four areas of theoretical knowledge about universal design identified among the participants, corresponding to the four subthemes: knowledge about digital barriers; knowledge about assistive technology; knowledge about the universal design concept; and knowledge about regulations, guidelines and standards.

#### Knowledge about digital barriers

Of the 35 participants, 30 share comments related to barriers. Of these, 28 have some knowledge about barriers, while two have no knowledge. Of the 28 participants with knowledge of barriers, 15 also have knowledge of both assistive technology (AT), including other aids, and digital barriers. Only 14 of the 30 participants state that they do not have any knowledge about this or they are unsure. Seven of these 14 participants with no knowledge of this topic also say they have little or no previous experience of working with students or others with disabilities.

##### Types of barriers

We have grouped the digital barriers the participants mention into four main categories: perceiving; operating; understanding and language; and other barriers. The three first categories can to some extent be related to the WCAG principles. An overview of the four main categories is given in Table 2 below, along with the number of participants who described barriers in each category. Note that some participants comment on more than one type of barrier.

**Table 2:** Overview of types of barriers

Type of barrier	Examples	Number of participants mentioning barrier
Perceiving	Hearing and/or seeing lectures, instructions, learning materials, physical and digital learning environment	19 in total, of these: 1 only auditory 11 only visual 7 both auditory and visual
Operating	Equipment, software, devices	9
Understanding and language	Spoken and written language, diction	9
Other barriers	Related to software, devices, compatibility	4

*Perceiving* includes barriers related to hearing or seeing lectures, instructions, learning materials, and the physical or digital learning environment. Barriers to perceiving are mentioned by 19 participants. Among these, 11 reveal knowledge on visual barriers, two on auditory barriers, and seven participants show knowledge on both visual and auditory barriers:

*“Not being able to see or hear may be a problem, because the courses are not prepared for that.”*

The auditory barriers mentioned among participants include not being able to hear oral lectures, instructions and explanations given while writing on the blackboard/whiteboard, sound in videos, and difficulties arising from people talking too fast or issues arising from diction. The only digital barrier mentioned is related to a lack of video captioning:

*“For deaf persons, […] video instructions, then just use captions.”*

Visual barriers are related to perceiving visual content. Among the digital barriers most often mentioned are inaccessible learning materials, such as PDF documents, lecture slides, images in presentations and small font size. Several also mention difficulties with not being able to see visual interfaces on laboratory equipment, e.g. oscilloscopes or electronic circuit boards. Only very few comment on issues related to the learning platform, for example faculty members publishing inaccessible learning materials (documents, videos), and that the learning management system lacks options for enabling settings that would improve accessibility, e.g. high contrast mode, enlarged letters or a different background colour. Other barriers mentioned include reflection and low contrast due to light (in the room) shining on the screen, systems with screens that cannot scale up, difficulties with not being able to see mathematical formulas or what is written on a blackboard/whiteboard, and issues with foreground and background colours. The following examples given by participants concern visual barriers:

*“We would have to help them [blind persons] to illuminate a lot of the visual interfaces and make the interfaces easier to navigate.”*

*“We can easily present a text, convert it to voice, but when we have advanced mathematical formulas, some engineering equations and so on, it becomes more tricky.”*

*“Electronics is visual stuff. It's relatively hard to imagine the structure of the circuit, and also the reading materials, because most of the materials are documentation. They could be transferred to braille, but the description of figures, which is the main content of the documentation, would probably be hard.”*

*Operating barriers* are related to being able to operate equipment, software and devices used in classes, as well as non-digital barriers related to practical tasks or tasks that require movement and barriers to participating in more practical courses such as drawing or design (as opposed to more theoretical courses). Although nine of the participants show some knowledge of potential barriers related to operating equipment or devices used in class or laboratories, only a few of the barriers mentioned are digital barriers:

*“It’s just different for blind people, they can listen. [...] The theoretical courses are probably easier to be transferred, the practical can be more difficult, like drawing or design.”*

*“Courses with lots of drawing were difficult, for instance data base design.”*

*Understanding and language* involves barriers to understanding the content of lectures, tasks and assignments, written materials, spoken and written language, and issues related to a faculty member’s dialect or diction, as well as issues related to students’ familiarity with the language used in the course (e.g. English). Nine of the participants have knowledge of barriers relating to understanding and/or language, and most of the barriers mentioned are not directly related to digital accessibility:

*“When a student doesn't know the language, for example English, and knows only some other foreign language.”*

*Other barriers* mentioned include using different software, formats and devices, and compatibility (formats, software, devices), which can all contribute to them experiencing difficulties in accessing digital content. Four participants have some knowledge of this group of barriers:

*“The problem is that some of the tools you have to use might not be designed universally.”*

*“A lot of students don't have access to PowerPoint, because they haven’t updated the program.”*

#### Knowledge about assistive technology

Of the participants, 24 mention different kinds of assistive technology (AT), while 19 express knowledge about other kinds of assistive aids (including human assistance). Both AT and other assistive aids are mentioned by 17 of the participants.

##### Types of AT

Table 3 gives an overview of the different types of assistive technologies that participants mention by name, or show they have some knowledge of, e.g. by describing it or using a different word than the common term. Note that some participants mention AT for both input and output, and some more than one type of AT as input or output.

**Table 3:** Types of assistive technologies of which participants indicated some knowledge

	Type of AT mentioned by participants	Number of participants
Output	Screen readers/text-to-speech	15
	Magnifying/scaling software	3
Input	Brain-computer interface	5
	Eye steering/control	5
	Voice recognition/speech-to-text	6
	Alternative to mouse	3

Not all participants see the need for assistive technologies, since they do not have any students who need this type of tool. Others comment that they are aware of systems that do not have any built-in assistive technologies being used in their courses:

*“I don’t think we have any students who need special equipment.”*

*“To be honest I didn't see anything in the system […] that would help […] people with disabilities to overcome the barriers.”*

Only two participants seem to be aware of the built-in assistive technologies available in operating systems:

*“I think Windows currently gives you a lot of options in terms of computer-based works for hearing impaired people.”*

*“There are some tools in every operating system that make it easier to operate, even some simple settings for blind people.”*

The results show that 18 individuals have some knowledge of assistive technologies for output, 15 of which have knowledge of technology for visual assistance, although not all of them use an appropriate term for this such as “screen reader” or “text-to-speech”. Some say they are impressed with this type of software and what it can do, while others express concerns that certain types of content, such as graphics and images, may not be adequately conveyed with these tools. Software for magnifying or scaling what is shown on the screen is mentioned by three participants, specifically enlarging the screen (contents) or scaling text or font:

*“Blind people use screen-readers, and are actually quite fast at doing this. … The descriptions under the pictures are important.”*

*“When it is text-related, text-to-speech can be a solution.”*

*“[…] some solutions to present, also images, to people with disabilities. I think they [the computers] can also interpret the information, the visual information, but I'm not aware of how this can be supported.”*

*“Someone not seeing well – then use some software that enlarges the screen/fonts.”*

Assistive technologies for alternative input that the participants mention include brain-computer interfaces, controlling the computer with the eyes such as through eye blinks or eye gaze, assistive tools involving voice recognition or speech-to-text, and alternatives to a mouse including touch interface, joysticks and special software. Knowledge about input technologies is described by 19 participants:

*“Some kind of brain-computer interface, so they can think about something and interact even if they are fully paralyzed.”*

*“There are solutions that allow you to control a computer with, for example, eye blinks.”*

*“When you have, e.g., deaf students and need to find some way to present explanations in a text format, then a solution could be speech-to-text systems.”*

*“It would probably be possible if we can replace traditional mouse-based input with something else, e.g., a joystick.”*

In addition, many show some awareness of assistive technologies albeit without being able to name or clearly explain any such tool or give coherent examples. Among these participants are also some who consider it necessary to create special solutions or adaptations for individuals or groups.

##### Types of other relevant assistive aids (not strictly AT)

Nineteen of the participants demonstrate some knowledge of assistive aids that can be used with computers or for accessing digital content, for example using Braille, software or services such as translation tools, writing aids, spell checkers and digital aids for exams, or physical items such as a bigger screen or a mouth stick stylus:

*“We will need to somehow redesign or rebuild boards/hardware, so all information will be available by touching/by braille information.”*

*“They also use writing aid programs, that help them to write.”*

*“I know that some use translations; Google translate and a lot of other things.”*

*“[…] they have computers with a spell-checker.”*

#### Knowledge about the universal design concept

Eighteen participants know nothing about the concept of universal design:

*“Universal design. I've heard of it, but I don't know in particular what's behind it.”*

A further 15 have superficial understanding of the concept. Some mentioned “accessible for everyone” or “for all groups of people”, people with disabilities, and the different ways of interacting with computers. However, they also admit to having little knowledge about the concept:

*“I've heard of the term, I think it's trying to design something that is accessible for everyone.”*

*“I think it's a design for everyone/for the people; young, older, with disabilities and… A sort of design that everyone should use.”*

*“That means that everybody, with all different disabilities, should be able to use an IT system, or other systems.”*

Only two participants have good knowledge about the concept:

*“What I like most about the term "universal design" is the idea that technology is a way of really democratising everything. It makes everything accessible for everyone and then if you say that the internet provides access to information, all the information in all the world, it needs to be accessible to all persons in all the world, and needs to be accessible through technology. To actually make it useful for everyone is thus a very big aspect of that.”*

When it comes to the principles of universal design, none of the participants were able to name any of the seven principles [50].

#### Knowledge about regulations, guidelines and standards

Out of the 35 participants, 18 know nothing or very little about regulations at the European level and 20 know nothing or very little about the UN CRPD. At the national level, 29 out of 35 know nothing or very little about relevant regulations. Those with very little knowledge only know about the universal design of buildings. One participant has heard of web accessibility. Only one of the 35 participants has good knowledge about regulations at both the national and international level:

*“The United Nations talks about systems, services and environments to be used for as broad a share of the population as possible, to the greatest extent possible without specialised adaptations[...] Norway is the only country that has a law about universal design and their definition is very congruent with the US, but Norway chose to regulate universal design using a web-accessibility standard as part of their approach to the universal design of ICT*.”

When it comes to W3C guidelines for web accessibility, authoring tool accessibility etc., 19 of 35 participants respond that they know nothing or very little about them. Those who know little have heard of them and are aware that these guidelines exist, but are not able to use them. Two participants can give a few random examples, such as colour contrast and user feedback, but none are able to explain them in a systematic manner:

*“I don’t think that the standards are well known and recognised. So, this is just a question of making designers of the contents and so on aware of some standards.”*

*“You have the WCAG 2.0. I know that that exists. I would be able to read about them, but I would not be able to use them as in programming something. Again, I haven't got that type of background, but I am aware of its existence.”*

Only two participants have good knowledge of WCAG, one of whom could explain its drawbacks:

*“As long as you follow WCAG, which is a standard about accessibility, and really it's not even accessible for [all] people with disabilities, because it's really about accessibility for people with sensory and some motor disabilities or physical disabilities. It's certainly not a standard that deals with cognitive disability to any real extent, then you automatically, kind of narrow the definition much further than it's conceived to be.”*

### Practical knowledge about how to make learning material accessible

In this section, we have attempted to concretise the results and make them more generally valid by mapping the solutions mentioned by the participants to the pure technical success criteria set out in WCAG 2.0. Hereby, the participants’ verbal expressions are interpreted to convey a glimpse of their practical knowledge of universal design of ICT. We conclude this section with a short presentation of the participants’ practical knowledge of individual accommodation.

#### Following WCAG 2.0

Of the 35 participants, 13 mention technical solutions for removing ICT barriers that are related to principles, guidelines and success criteria in WCAG 2.0. They cover all four principles, 8 of the 12 guidelines and 14 of the 61 success criteria. The 13 participants exemplify between one and four success criteria each. The Norwegian Regulation for universal design of information and communication technology (ICT) solutions currently require fulfilment of 35 of the 61 success criteria in WCAG 2.0 [51].

Three participants appeared to have better knowledge about technical solutions that make learning materials accessible for most students, since they gave more examples and more detailed explications than the other participants:

*“If you use images in your presentations, you need to explain the images if they’re necessary, if they’re just there for decoration then you might not need to.”*

Many participants clearly state that they do not have practical know-how:

*“My PDF, I guess, can’t be made available for blind people. I don’t know how that can be done.”*

*“Maybe just listening to the voice or… I don't know if graphics are possible to describe.”*

The principles and success criteria in WCAG 2.0 that 13 of the participants were aware of are shown in Table 4. The levels of conformance have not been included in this table, but are specified in the section describing each principle.

**Table 4:** Overview of all WCAG principles and success criteria the participants are aware of

Principles	Success criteria	Participant themes	Number of participants
1. Perceivable	1.1.1	Alternative text	4
	1.2.2	Captions	2
	1.2.8	Media alternative	1
	1.3.2	Meaningful sequence	1
	1.3.3	Sensory characteristics	2
	1.4.1	Use of colour	1
	1.4.3	Contrast (minimum)	3
	1.4.4	Resize text (200%)	3
2. Operable	2.1.1	Keyboard	4
	2.4.1	Bypass blocks	1
3. Understandable	3.2.3	Consistent navigation	1
	3.2.4	Consistent identification	1
4. Robust	4.1.1	Parsing	2
	4.1.2	Name, role, value	2

##### Principle 1: Perceivable

Four guidelines are covered under this principle, and eight success criteria on level A (5 participants), AA (2) and AAA (1), of 22 success criteria in total, were exemplified by 10 participants. The most frequent examples are related to alternative text (4), contrast and zoom (3), and captions and sensory characteristics (2):

*“Every image, every graphic has to have a text description; an alternative text… a way to explain it to somebody who can’t see it.”*

*“You need bigger fonts, more contrast (in the learning management system).”*

*“It should be possible to submit the contents in a way that is accessible not just in one possible way.”*

*“I use a lot of […] video captioning.”*

##### Principle 2: Operable

Two of four guidelines under this principle are covered, and two success criteria on level A, of a total of 20 success criteria on level A, AA and AAA, are exemplified by four participants. The example below applies to keyboard accessibility and simple navigation:

*“Make the interfaces easier to navigate using a keyboard, so they don't have to tab through a whole list of menus until they can get to the correct link.”*

##### Principle 3: Understandable

One of three guidelines under this principle is covered, and two success criteria on level AA, of a total of 17 success criteria on level A, AA and AAA, are exemplified by two participants. The examples below concern consistent navigation and identification:

*“Design of web pages that make them easy to read.”*

*“Follow practices on how to structure the information and be precise on where you're giving information, so that it's predictable for everyone.”*

##### Principle 4: Robust

The one guideline under this principle, with two success criteria on level A, is covered. The examples given by two participants apply to compatibility with different devices and assistive technologies:

*“Being able to open stuff in different tools, mobiles, tablets, that kind of thing.”*

*“Making it accessible for screen readers and things like that.”*

#### Individual accommodation

All participants had suggestions on how to make individual accommodations for diverse students. However, only two participants had examples of individual digital solutions, and these were related to the development of special interfaces:

*“We can provide more adjusted interfaces – adapted, suitable interfaces for people with disabilities.”*

The individual non-digital solutions described by the participants are mainly related to the following categories: Additional time with students, Student assistance, Braille and Embossed materials**,** Written communication and Audio recordings.

Assistive technologies, mentioned as an individual solution for removing ICT barriers, are not presented here, even though they might be digital, since faculty members do not usually have responsibility for such accommodations. However, ATs are covered in Sect. 5.1.2 above.

## Discussion

The results from the thematic analysis show that, in general, there is a lack of sufficient understanding of digital barriers and assistive technologies. Very few are aware of legislation and guidelines related to universal design. Most importantly, the majority lacked practical knowledge on how to make digital learning materials and courses accessible. Our findings are in accordance with previous studies [e.g. 19–24].

### Theoretical knowledge

One of the obstacles to achieving equal participation in higher education is that many faculty and administrative staff have limited knowledge of legislation relating to disability rights and of appropriate accommodations for students with disabilities in the classroom [52]. In their focus group interviews, Burgstahler et al. [53] found that most faculty knew very little about their legal responsibilities and relied on student support services for information, although they wanted to learn about laws and regulations, specific disabilities (particularly learning and other invisible disabilities) and how to accommodate students. Our study, carried out 20 years after both Leyser et al. [52] and Burgstahler et al. [53], has very similar findings, indicating that the situation has not improved very much.

In terms of potential barriers for students, the participants often talked about non-digital barriers, particularly architectural barriers such as lab space and interior design, as identified by Jeannis et al. [35]. It is a general understanding that architectural barriers are more visible and easier to recognise and exemplify than those in the digital environment. In addition, the participants, as faculty members of university courses, consider the perceived barriers, mostly architectural, as “problems” that may prevent students from attending their courses. This indicates a lack of awareness and knowledge of digital barriers and terminology among the participants for talking about such barriers, as well as a shallow understanding of the consequences they entail for the students.

As regards assistive technology, many participants seem to believe that having assistive technology is sufficient for students with disabilities to access digital learning materials, and that they, as the creators of the learning materials, do not need to do anything more. Such belief indicates a lack of knowledge and understanding that, for assistive technology to be useful, digital learning materials should be universally designed. It is not necessary that faculty members have thorough knowledge of assistive technologies or whether a student uses braille or switch input. However, it is an advantage for faculty to be aware of these technologies, which can help them to understand why they should make their digital materials accessible.

### Practical knowledge

In order to make learning materials accessible, faculty members need practical know-how. The participants in our study have shown a general willingness for individual accommodation, for example by providing more time or specific solutions for individual students. However, they lack practical knowledge on how to make their digital material accessible to all students. Examples of essential knowledge we found to be lacking among the participants include using built-in styles for structure, unique and understandable link text, colour and contrast, and subtitles in videos.

Through the analysis, we found that the participants could give examples of what accessible digital learning materials should be like, e.g. providing captions for videos, increasing the font size, describing images and supporting navigation by keyboard. This shows that they know some accessibility success criteria covered in the Web Content Accessibility Guidelines (WCAG), although they may not have used that specific terminology. We found that very few participants were able to use appropriate terminology to describe barriers and their respective solutions. A lack of terminology when talking about digital barriers as well as accessibility-related standards and guidelines indicates that the participants need relevant training.

### Implications

Making digital learning material accessible requires practical knowledge, such as using heading styles to structure documents, providing captions for videos and providing text descriptions for images. Although theoretical knowledge such as the concept of universal design, relevant legislation, accessibility standards and digital barriers does not directly provide the knowledge required for making learning material accessible in practice, it helps faculty members to understand why making digital material accessible is necessary and what legislation and standards apply to their work.

In our study, we found that the more knowledgeable participants had gained their knowledge through experience with people with disabilities. In Burke and Sutherland [54], a statistically significant relationship was found between prior experience and knowledge of people with disabilities and attitudes toward inclusion. We argue that training faculty members to be more knowledgeable about digital inclusion would lead to more positive attitudes and better services for diverse students.

The solutions proposed by nearly all of the participants were to allocate time to speak with students who experience barriers and can suggest solutions. Consequently, it becomes the students’ responsibility to take the initiative to contact faculty members, and it is thus up to the faculty members to provide individual accommodation on a case-by-case basis rather than providing universally designed solutions. As seen in the accessibility pyramid (Fig. 1), individual solutions represent the second highest level of accommodation (level 3). However, the universal design of digital learning materials (level 1) reduces the need for individual accommodation and would benefit most students. To fulfil the requirements set out in the UN CRPD and national legislation, HEIs should have policies that include procurements, workflow guidelines and clearly stated requirements for ensuring accessible digital learning materials.

Langørgen et al. [46] also found a lack of knowledge on how to accommodate students, time constraints and insufficient institutional support in their study exploring the perspectives of academic staff and placement supervisors on supporting students with disabilities in professional study programmes within health care, social work and education. In line with our study, this demonstrates a continued need to increase knowledge among faculty members.

Hartsoe and Barclay’s [45] findings show a more hopeful picture demonstrating the commitment of a growing number of faculty members in higher education to provide course materials in a way that is accessible. In the meantime, the authors also recognise that there is still room for HEIs to grow in attaining a higher level of accessibility [55–57], and increasing training in faculty preparation programmes could boost the accessibility of instruction.

Our study also indicates that in order to provide training and support to faculty members, institutions should have policies and strategies for implementation, and allocate resources to this end. This is in accordance with other studies [e.g. 24, 28], which emphasise the importance of institutional policy, investment and responsibilities in coordination, monitoring and evaluation when implementing inclusive education.

### Limitations

There are three major limitations to this study that could be addressed in future research. Firstly, the participants were recruited from computer science and engineering faculties, an approach also employed in other studies [e.g. 19, 23], and our participants represented a small number of higher education institutions in Norway and Poland. Although we sent invitations to a wide range of HEIs and have interviewed all the 35 persons that responded to our invitations, the convenient sampling strategy and the limited number of participants have resulted in a lack of representativeness, which may have further caused bias and negatively affected the reliability and generability of the results. During the data analysis process, we found that participants from the same institutions responded to some of the questions in a similar manner. Such limitation may prevent us from generalising the findings. Nevertheless, we would argue that faculty members in computer science and engineering faculties are expected to have a higher level of digital competence than those from other disciplines, and are therefore in a better position to make their learning materials accessible. To address this limitation, future research should consider a combination of qualitative and quantitative methods and include more participants representing a wider range of faculties and institutions.

Secondly, this paper presents a qualitative study where semi-structured interviews were used to collect data. Although the semi-structured interviews allowed participants to express their thoughts and reflect on their experience, it also limited the possibility of obtaining precise data on their knowledge in practice. We instead had to rely on our interpretation of their statements to understand their level of knowledge, which could have introduced bias. We are aware that we may have interpreted the participants’ knowledge in a more positive light than was in fact the case. Asking the participants to demonstrate their practical know-how and observing their knowledge in practice would have allowed us to gain a more objective understanding of their practical knowledge.

Thirdly, although the data was collected in two countries with different legislation concerning the universal design of ICT, we have chosen to look at the dataset as a whole and not to compare the knowledge level of the participants between the two countries due to the qualitative nature of the study and the limited number of participants. A comparative analysis of the differences in knowledge between the two countries in relation to their respective legislation may provide a richer understanding of the factors and context that affect knowledge. Such an analysis will also have implications for policymaking related to the universal design of ICT in both countries. Future research should collect quantitative data from a representative sample of HE faculty in both countries for a comparative analysis in order to provide a richer understanding of their knowledge and contributing factors.

## Conclusion and future work

In order to ensure diverse students equal access to higher education, it is important that teaching staff have knowledge of how to avoid digital barriers and make teaching and learning materials inclusive.

In this research, we have investigated knowledge of universal design and digital accessibility among faculty members in the fields of computer science and engineering. The findings show that very few participants are knowledgeable in this respect, particularly on how to make digital learning material and courses accessible.

The research demonstrates the pressing need to provide training in universal design and digital accessibility for faculty members. This need is even more prevalent during the COVID-19 pandemic when students, faculty and staff with disabilities at universities have faced many digital accessibility barriers [58]. In order to gain such knowledge, faculty members need time to learn and practice. This further requires higher education institutions to support these efforts by allocating time and resources to this end as part of professional development for faculty members. In order to be effective, such support should also be prioritised in the policies, strategies and action plans of the institutions.

Following the conduct of our study in 2016, there have been developments in the legislation in both countries and in the EU. In Norway, in 2018, an enactment to the regulation related to Sect. 18 of the Norwegian Equality and Anti-Discrimination Act required electronic learning platforms and digital learning materials used in education in Norway be universally designed [36]. In Poland, The Law of Higher Education was replaced by the Law of Higher Education and Science that came into force in 2018. In 2018, the Polish governmental programme Accessibility Plus 2018–2025 was launched, aiming to improve accessibility in all areas of society, including education [59]. The EU Directive from 2019 on the accessibility requirements for products and services [60] does not apply to the educational sector. Despite the positive changes in the legislation and the promising governmental programmes, our findings indicate that there is a gap between legislation and implementation in practice at institutional level when it comes to making digital learning materials accessible in higher education. Therefore, it is important that institutions stipulate requirements for and carry out monitoring and evaluation of the implementation of inclusive education.

## Appendix: Interview guide

Interview guide/questions for the semi-structured interview.

- What subject do you teach? How many years have you taught the subject?
- Do you have experience with teaching diverse students? If not, move on. If yes, ask how.
- Imagine you have a student with a disability (e.g. blind, motor skills). Do you know how to help him/her?
- Are you aware of the diverse students who have difficulties in accessing ICT systems?
- What barriers do you think that the current ICT systems have for everybody in education? (learning material, learning environment, assistive technology)
- What do you think would be the solution for removing the barriers?
- Do you know anything about the term universal design? (if not, explain UD) (if yes, ask whether they are aware of the standards/principles)
- (Leading to education) Do you think including universal design principles in curricula would be useful?
- Would you be willing to include UD principles in your own curricula?
- Can you see any challenges in including UD principles in your own curricula? If so, what?
- Are you aware of the UN CRPD Article 9?
- What do you think about the relevant legislation in your country?
- Any additional comments?

## References

[1] Hauschildt, K., Vögtle, E. M., Gwosć, C.: Social and economic conditions of student life in Europe. EUROSTUDENT VI 2016–2018 | Synopsis of Indicators. Table B 1.5. German Centre for Higher Education Research and Science Studies (DZHW) (ed). (2018) 10.3278/6001920cw

[2] Kent M (2015). Disability and eLearning: opportunities and barriers. Disabil. Stud. Q..

[3] Sachs D, Schreuer N (2011). Inclusion of Students with disabilities in higher education: performance and participation in student’s experiences. Disabil. Stud. Q..

[4] Seale J (2013). When digital capital is not enough: reconsidering the digital lives of disabled university students. Learn. Media Technol..

[5] European Commission (2010) European Disability Strategy 2010–2020: European Disability Strategy 2010–2020: a renewed commitment to a Barrier-Free Europe. Retrieved from http://eurlex.europa.eu/LexUriServ/LexUriServ.do?uri=COM:2010:0636:FIN:en:PDF

[6] Chen, W., Sanderson, N. C., Kessel, S. (2018) Making Learning materials accessible in higher education—attitudes among technology faculty members. In: Craddock, G., Doran, C., McNutt, L., Rice, D. (Eds.) Transforming our world through design, diversity and education. Proceedings of Universal Design and Higher Education in Transformation Congress 2018. Studies in Health Technology and Informatics. 10.3233/978-1-61499-923-2-87 30371463

[7] Haugeto, Å. K.: Trend Spotting at UD2012Oslo. In: Trends in Universal Design. Norwegian directorate for children, youth and family affairs (Bufdir), The Delta Centre, pp 6–9 (2013), ISBN 978–82–8003–101–3

[8] Centre of Excellence in Universal Design (CEUD) (n.d.): What is Universal Design. http://universaldesign.ie/What-is-Universal-Design/ Accessed 15 June 2020

[9] Equality and Anti-Discrimination Act: Act relating to equality and a prohibition against discrimination (LOV-2017–06–16–51). (English version). (2017) https://lovdata.no/dokument/NLE/lov/2017-06-16-51

[10] Stephanidis C (2001). User interfaces for all.

[11] Moseid, T. E.: Mind the gap! Library services to the disabled in a new framework. LIBREAS. Library Ideas, 6 (2006). Retrieved from https://libreas.eu/ausgabe6/002mos.htm

[12] Nygaard, K. M.: What is universal design – theories, terms, and trends. IFLA World Library and Information Congress (IFLA WLIC) (2018). Retrieved from http://library.ifla.org/2250/1/094-nygaard-en.pdf

[13] Nordby, K.: eAccessibility for all: the role of standardisation in shaping the end-users’ tel-eEurope. In: Lehne, P. H. (Ed.) Teletronikk, 100 (1):4–13, (2004), ISSN 0085–7130. Retrieved from http://www.telenor.com/wp-content/uploads/2012/05/T04_1.pdf

[14] Fuglerud, K. S.: Inclusive design of ICT: the challenge of diversity. Dissertation, University of Oslo (2014) 10.13140/2.1.4471.5844

[15] World Wide Web Consortium: Web content accessibility guidelines (WCAG) 2.0. Caldwell, B., Cooper, M., Guarino Reid, L., Vanderheiden, G. (Eds.) W3C Recommendation 11 December 2008. http://www.w3.org/TR/WCAG20/ Accessed 15 June 2020

[16] World Wide Web Consortium: Web Content Accessibility Guidelines (WCAG) 2.1. Kirkpatrick, A., O Connor, J., Campbell, A., Cooper, M. (Eds.) W3C Recommendation 05 June 2018. https://www.w3.org/TR/WCAG21/ Accessed 15 June 2020 (2018)

[17] International Organization for Standardization: Web Content Accessibility Guidelines (WCAG) 2.0, (ISO/IEC Standard No. 40500:2012), (2012) Retrieved from https://www.iso.org/standard/58625.html

[18] United Nations: The UN Convention on the Rights of Persons with Disabilities (CRPD). (2006) Retrieved from https://www.un.org/development/desa/disabilities/convention-on-the-rights-of-persons-with-disabilities.html

[19] Ratifications and Signatures of the UN CRPD (n.d.): https://treaties.un.org/Pages/ViewDetails.aspx?src=TREATY&mtdsg_no=IV-15&chapter=4&clang=_en Accessed 4 March 2021

[20] Soriano, V., Watkins, A., Ebersold, S.: Inclusive education for learners with disabilities. European Union (2017). Retrieved from https://www.europarl.europa.eu/committees/en/supporting-analyses/sa-highlights

[21] Hériard, P.: Higher Education. Fact Sheets on the European Union. Retrieved from https://www.europarl.europa.eu/factsheets/en/sheet/140/higher-education Accessed 5 March 2021

[22] Directive (EU) 2016/2102 of the European parliament and of the Council of 26 October 2016 on the accessibility of the websites and mobile applications of public sector bodies. Retrieved from https://eur-lex.europa.eu/eli/dir/2016/2102/oj Accessed 5 March 2021

[23] Poland in the EU (n.d.) Website of the Republic of Poland. https://www.gov.pl/web/eu/poland-in-the-eu Accessed 12 March 2021

[24] European Economical Association (EEA) (n.d.) https://www.efta.int/eea Accessed 8 March 2021

[25] European Free Trade Association (EFTA) (n.d.) https://www.efta.int/eea/eea-agreement Accessed 8 March 2021

[26] Meld. St. 5 (2012–2013) Report to the Storting (White Paper). The EEA Agreement and Norway’s other agreements with the EU. Retrieved from https://www.regjeringen.no/globalassets/upload/ud/vedlegg/europa/nou/meldst5_ud_eng.pdf

[27] Anti-Discrimination and Accessibility Act (No. 42 of 2008). Available at https://www.ilo.org/dyn/natlex/natlex4.detail?p_lang=&p_isn=88353

[28] Regulations for universal design of information and communication technology (ICT) solutions (2013). Retrieved from https://www.regjeringen.no/en/dokumenter/regulation-universal-design-ict/id731520/

[29] Norwegian Act relating to universities and university colleges (LOV-2005–04–01–15) (2004) Section 4–3.2. Available at https://lovdata.no/NLE/lov/2005-04-01-15/§4-3

[30] Moroń, D.: The higher education of people with disabilities in Poland. Scientific and Methodical Review: Education for Safety VII (4/2014 (25)):215–227 (2014) Retrieved from https://www.academia.edu/download/38531739/pasja_gisw.pdf#page=215

[31] Shinohara, K., Kawas, S., Ko, A. J., Ladner, R. E.: Who Teaches Accessibility? Paper presented at the Proceedings of the 49th ACM technical symposium on computer science Education - SIGCSE '18 (2018)

[32] Marquis E, Jung B, Fudge-Schormans A, Vajoczki S, Wilton R, Baptiste S, Joshi A (2012). Creating, resisting or neglecting change: exploring the complexities of accessible education for students with disabilities. Can. J. Scholarsh. Teach. Learn.

[33] Marquis E, Jung B, Fudge Schormans A, Lukmanji S, Wilton R, Baptiste S (2016). Developing inclusive educators: enhancing the accessibility of teaching and learning in higher education. Int. J. Acad. Dev..

[34] Marquis E, Fudge Schormans A, Jung B, Vietinghoff C, Wilton R, Baptiste S (2016). Charting the landscape of accessible education for post-secondary students with disabilities. Can. J. Disabil. Stud..

[35] Jeannis H, Goldberg M, Seelman K, Schmeler M, Cooper RA (2019). Barriers and facilitators to students with physical disabilities’ participation in academic laboratory spaces. Disabil. Rehabilit. Assist. Technol..

[36] Proba Research: Universell utforming av IKT med vekt på læremidler i UH-sektoren. Rapport 2019–02. ISSN: 1891–8093. (English title: Universal Design of ICT emphasizing teaching aids in the Higher Education sector) (2019)

[37] Langørgen E, Magnus E (2018). ‘We are just ordinary people working hard to reach our goals!’ Disabled students’ participation in Norwegian higher education. Disabil. Soc..

[38] Cook L, Rumrill PD, Tankersley M (2009). Priorities and understanding of faculty members regarding college students with disabilities. Int. J. Teach. Learn. High. Educ..

[39] Holloway S (2001). The experience of higher education from the perspective of disabled students. Disabil. Soc..

[40] Linder KE, Fontaine-Rainen DL, Behling K (2015). Whose job is it? Key challenges and future directions for online accessibility in US Institutions of higher education. Open Learn. J. Open Distance e-Learn..

[41] McManus JL, Feyes KJ, Saucier DA (2010). Contact and knowledge as predictors of attitudes toward individuals with intellectual disabilities. J. Soc. Pers. Relat..

[42] Fabrigar LR, Petty RE, Smith SM, Crites SL (2006). Understanding knowledge effects on attitude-behavior consistency: the role of relevance, complexity, and amount of knowledge. J. Pers. Soc. Psychol..

[43] Brillhart BA, Jay H, Wyers ME (1990). Attitudes toward people with disabilities. Rehabil. Nurs..

[44] Leyser Y, Greenberger L (2008). College students with disabilities in teacher education: faculty attitudes and practices. Eur. J. Spec. Needs Educ..

[45] Hartsoe JK, Barclay SR (2017). Universal design and disability: assessing faculty beliefs, knowledge, and confidence in universal design for instruction. J. Postsecond. Educ. Disabil..

[46] Langørgen E, Kermit P, Magnus E (2018). Gatekeeping in professional higher education in Norway: ambivalence among academic staff and placement supervisors towards students with disabilities. Int. J. Incl. Educ..

[47] Hill S, Roger A (2016). The experience of disabled and non-disabled students on professional practice placements in the United Kingdom. Disabil. Soc..

[48] Black RD, Weinberg LA, Brodwin MG (2014). Universal design for instruction and learning: a pilot study of faculty instructional methods and attitudes related to students with disabilities in higher education. Excep. Educ. Int..

[49] NSD - Norwegian centre for research data (n.d.) https://www.nsd.no/en

[50] Centre of Excellence in Universal Design (CEUD). (n.d.). The 7 Principles. http://universaldesign.ie/What-is-Universal-Design/The-7-Principles/ Accessed 15 June 2020

[51] Forskrift om universell utforming av informasjons- og kommunikasjonsteknologiske (IKT)-løsninger (2013) (FOR-2013–06–21–732). (English: Regulation for universal design of information and communication technology (ICT) solutions.) Retrieved from https://lovdata.no/dokument/SF/forskrift/2013-06-21-732 Accessed 15 June 2020

[52] Leyser Y, Vogel S, Wyland S, Brulle A (1998). Faculty attitudes and practices regarding students with disabilities: two decades after implementation of Section 504. J. Postsecond. Educ. Disabil..

[53] Burgstahler, S., Duclos, R., Turcotte, M. (2000) Preliminary findings: faculty, teaching assistant, and student perceptions regarding accommodating students with disabilities in postsecondary environments. University of Washington, Seattle DO-IT. (ERIC Document Reproduction Service No. ED456718)

[54] Burke K, Sutherland C (2004). Attitudes towards inclusion: knowledge vs experience. Education.

[55] McGuire JM (2014). Universally accessible instruction: Oxymoron or opportunity?. J. Postsecond. Educ. Disabil..

[56] McGuire JM, Scott SS, Shaw SF (2003). Universal design for instruction: the paradigm, its principles, and products for enhancing instructional access. J. Postsecond. Educ. Disabil..

[57] McGuire JM, Scott SS (2006). Universal design for instruction: extending the universal design paradigm to college instruction. J. Postsecond. Educ. Disabil..

[58] Lazar J (2021). Managing digital accessibility at universities during the COVID-19 pandemic. Univers. Access Inf. Soc..

[59] The Ministry of Investment and Economic Development: Governmental programme Accessibility Plus 2018–2025 (2018). Retrieved from https://www.funduszeeuropejskie.gov.pl/media/72628/Dostepnosc_angielski.pdf

[60] Directive (EU) 2019/882 of the European parliament and of the council of 17 April 2019 on the accessibility requirements for products and services. Retrieved from https://eur-lex.europa.eu/legal-content/EN/TXT/HTML/?uri=CELEX:32019L0882&from=EN Accessed 5 March 2021

